# Medical Society of the County of Albany, Semi-Monthly Meeting, Nov. 22d, 1875

**Published:** 1875-11

**Authors:** James S. Bailey


ART. III. *Medical Society of the County of Albany Semi-Monthly Meeting, Nov. 22d,* 1875. Reported by JAMES S. BAILEY, M. D.

Dr. HENRY MARCH, President, in the chair.

The President made a few happy remarks, after the reading of the minutes of the last meeting. He thanked the Society for the large majority they had given him in electing him President, and stated that he would do his part in keeping up an interest in the Society. He would adhere strictly to parliamentary usages, and urged the necessity of punctual attendance, and through discussion upon every question that might be presented.

Dr. QUACKENBUSH rose and said he was sorry that the first duty the President would have to perform should be a sad one, and he asked leave to present the following resolutions:

Whereas, we have heard with feelings of regret the announcement of the death of Frederick G., youngest son of Dr. John Swinburne, just entering upon early manhood, therefore,

*Resolved*, That this Society extend to our fellow associate and friend, Dr. Swinburne, our heart-felt sympathy in this hour of his bereavement and sorrow.

Adopted unanimously—the members standing.

Dr. QUACKENBUSH then proceeded to report a case of Atresia Vaginæ with treatment at time of delivery. He said, I propose to present to the Society a case of Astresia Vaginæ complicating delivery, and in describing the subject I will speak of the nature of Atresia, its causes and treatment, then describe the case and its management. Atresia, as its name signifies, is a non-perforated condition of the vagina, commonly called occlusion of the vagina. It may be congenital, when there is an entire absence of the canal, or it may be accidental, and then the obliteration may be partial or complete. Its causes are impaired or arrested developement, prolonged or difficult labor, chemical agents locally applied, mechanical injuries, and inflammations, the result of gonorrhœa or syphilis. When the occlusion is the result of inflammation in infancy or childhood, the seat of the adhesion is low down in the vagina, when the result of inflammation or sloughing consequent on protracted labor, it is more apt to be met with in the middle or upper third of the vagina; and in the cases which I have met with as the result of local applications, the occlusion has been near to, and involving the neck of the womb.

The treatment must vary with the nature of each case. When the adhesions are formed at the entrance of the vagina, the parts must be separated by an incision from top to bottom of the vulva, and the opening maintained by the insertion of a pledget of lint saturated with glycerine. When the adhesion is farther up in the vagina the parts must be drawn apart laterally, a groved director passed through the aperture, and following this a bistoury, by which incision must be made first on one side and then on the other, always carefully avoiding cutting above and in front or directly behind for fear of injuring the bladder in one case and the rectum in the other.

An important question here arises. How far shall the incision extend? Shall the whole opening be made with the knife? The best practice seems to be to divide the mucous membrane to a sufficient extent to allow of the entrance of the finger and then tear the opening till a sufficient enlargement is obtained. This procedure has two advantages; by means of tearing the parts we avoid the cutting of arteries, and according to some authorities, the parts are less likely to unite by adhesive inflammation after being torn apart than when divided by the knife.

The vagina should be cleaned by the injection of warm water, and then the aperture made should be kept open with a cylindrical pledget of lint saturated with glycerine and introduced through the entire length of the canal. Authors have written much about the vaginal walls contracting and again adhering but in my experience I have not found such to be the case. Having now given a definition of the abnormal condition of Atresia Vaginæ I will narrate a case:

Mrs. A——, æt. 23, was confined with her first child in 1867, in the city of New York. Her labor was protracted, and she was instrumentally delivered of a dead child. She did not become pregnant again till February last, and the tenth of this month she was taken in labor under the care of Dr. Newcomb of this city. The Doctor at once recognized the condition of the patient and invited me in consultation. Upon examination we found an almost entire occlusion of the vagina about two-thirds up the canal. The small aperture through which the menstrual discharge had escaped, would scarcely admit of a small director; through this aperture, the woman having been placed on the side of the bed in a suitable position for instrumental delivery—a director was inserted, and gradually a bistory was thus introduced and incisions made on either side. The opening was large enough to admit the introduction of two fingers. A further examination was made, and the womb being found open, the membrane protruding, and the pains strong and frequent, the sac was ruptured and the waters escaped. The forceps were now applied, the opening made in the vagina being sufficient to admit of the blades, and in a few moments a living child, weighing 10½ pounds, was delivered. Of course the walls of the vagina and consequently the opening made were much dilated during the delivery. The subsequent treatment by Dr. Newcomb has consisted in the introduction of pledgets of lint, thus keeping the parts well distended. The mother and child are both doing well. The Doctor showed the Society the forceps used by Dr. Newcomb, and stated he used them in perference to his own, as the opening made in the occulsion would more readily admit the blades. In speaking of chemical agents causing Atresia he said he referred to their use in the treatment of various troubles of the vagina. They were not the cause in this case. The treatment must depend on the peculiarities of each case. In one on which he operated before the patient became pregnant, he repeated the incisions several times and carefully kept the vagina open. The patient afterwards became pregnant, and labor coming on she was delivered without any trouble, the labor being in every way natural. The Doctor referred to another which occurred in the City Hospital about ten years ago, Dr. March examined the patient but did not at first find any opening in the occluding membrane. Upon further examination a small opening was found, and using it as a guide, it was enlarged and delivery followed. Death, however, resulted from peritonitis. This case was thought by the Doctor to have been the result of gonorrhœa.

Another case which happened in the Doctor's practice was caused by the use of lunar caustic, and the patient stated when first seen that she had falling of the womb. On examination it was found to be occlusion of the vagina.

Dr. Blatner referred to a case reported last year by Dr. Ten Eyck, which he thought somewhat similar only the opening in the occlusion was smaller. Dr. Ten Eyck stated that it would only admit an eye probe. These cases are rare, and he could only find one reported, that was in the Lancet of 1834, and was a case of the late Dr. James McNaughton's. The literature on this subject is scanty, and judging from the way some authors treated the subject, they could not have seen many cases. Some authors mentioned the danger of injuring the peritoneum, but we know that the peritoneum does not cover the whole uterus and is necessarily pushed upward by the gravid organ.

The President asked if this was the first case Dr. Quackenbush had had? The Doctor replied that he had had four.

Dr. HAILES inquired if the occlusion was in the upper or lower two-thirds?

Dr. QUACKENBUSH said the upper two-thirds.

Dr. HAILES remarked that that simplified the operation having the bag of waters pushing the occlusion down. He also asked if in any of his cases the occlusion had been near the womb?

Dr. QUACKENBUSH said it had in the case mentioned as being in the City Hospital.

Dr. HAILES asked if the Doctor did not consider it remarkable that the spermatozoa should find their way through the small opening in the membrane. It was generally thought that the cilliated epithelium of the vagina was an important aid to the movements of the spermatozoa, but this case seemed to give more importance to the spermatozoa themselves?

Dr. QUACKENBUSH replied, Solomon says "There are three things which are too wonderful for me, yea four, which I know not, and one of them is the way of man with a maid."

Dr. DEVOL wished to know from Dr. Quackenbush or Dr. Newcomb, if pregnancy occurred after the occlusion or before?

Dr. QUACKENBUSH said he had no doubt the occlusion occurred at the time of her first confinement in 1867.

Dr. BLATNER thought the question was fully answered in Dr. Ten Eyck's case when the patient was examined before marriage, and the occlusion found to exist. The spermatozoa are the active principle in fecundation, and the result of these cases teach us an important point in a medico-legal view. It has been found by experiment that the spermatozoa will penetrate through a thin membrane by their own activity, and if they can pass through such a substance, without any opening, why not through a hole large enough to admit a probe.

Dr. DAVIS said in the case of congenital atresia in which he had operated, he could find no opening larger than a fine silver wire, and could only introduce that about half an inch. If this small canal extended to the uterus it was very tortuous. He had to cut very cautiously and slowly, and by careful dissection make his way to the uterus. The incision measured four and a half inches when he had finished. He could not tear the parts—they being so firm—he was unable to make any impression. Could only cut—tried to use a director but was unable to do anything with it.

With the sound in the bladder, his finger frequently introduced into the rectum, he worked his way with the knife to the uterus.

Dr. QUACKENBUSH enquired how Dr. Davis knew his case to be congenital?

Dr. DAVIS replied he could only judge from her history. She had never menstruated. At certain periods resembling monthly sickness, she suffered great pains, and sometimes when in this condition, by introducing her fingers into the vagina and pressing upon the occlusion she could press out a few drops of dark blood.

Dr. QUACKENBUSH asked if the Doctor had made any examination for the uterus.

Dr. DAVIS said in company with two others he had examined per rectum and felt the uterus high up. At the time of operating he reached the uterus, his knife suddenly opened into some cavity, and he thought he had cut into the rectum, but it prove to be the cavity of the cervix, considerably dilated, probably by continual pressure of menstrual fluid.

Dr. BIGELOW inquired whether the patient menstruated after the operation?

Dr. DAVIS answered she did regularly, but in very small quantities.

Dr. MOORE stated he had a case at present where the uterus is absent. Had one where the vagina was occluded by an imperforate hymen, and when operated on a large amount of menstrual fluid was released.

Dr. HAILES asked if it was not considered dangerous to operate for the removal of the whole retained menstrual secretions at one time. If it was not better to remove it by several operations?

Dr. QUACKENBUSH said he thought it was.

Dr. James P. Boyd, Jr., reported a case of empyema, and said the patient was 45 years of age, native of Germany, and by occupation a saloon keeper, came to St. Peter's Hospital to be treated for a diarrhœa which he said had troubled him for three months. He stated that previous to this time he had always been well, and attributed his condition solely to the diarrhœa. His appearance attracted my attention at once; he was very pale and emaciated, and his features were unusually sharp. After close questioning I found that for the last few weeks he had chills, drenching sweats, and some fever towards evening. His appetite had left him, and he was very weak. His bowels would be loose one day and constipated the next, and the dejections were small and thin. The family history was good. He complained of no pain, cough nor shortness of breath, and was irritated when I questioned him in regard to his chest. Said sarcastically that he never had a pain in his chest, and that if we should cut him open we would find his lungs perfectly sound. I examined the chest carefully and found on the left side from the lower third of the scapula downward absolute dullness, absence of respiratory murmur, and vocal fremitus, the line of dullness extended somewhat higher near the axilla. From the severity of the constitutional symptoms I inferred that the fluid in question was purulent. The next day the house physician informed me that his evening temperature was 102° F., pulse 100, and that in addition to the chill and sweats he complained of pain in the right inguinal region about an inch above Pouparts ligament. On examination I found a hard mass the size of a horse chestnut somewhat tender to the touch. He walked about the room without pain. The bowels were still loose, notwithstanding the measures taken to control them, and the patient said he had to force himself to eat. The following day I found him for the first time in bed, and the uneasiness in the inguinal region had increased greatly. He now complained of some pain in moving the right leg, and was unable to retain anything on his stomach. Towards evening the house physician was obliged to use a catheter to empty the bladder. During the next day the skin over the region of the cæcum became discolored, and the swelling was now plainly visible. The dullness in the region increased and extended to the brine of the pelvis, a discoloration was also visible below Pouparts ligament. He could not move the right leg, and vomited his food. The urine was again drawn off, his strength failed rapidly, and he died that night, remaining perfectly conscious up to the time of his death.

Autopsy 14 hours after death. Body much emaciated, skin discolored in right inguinal region and extending into right lumbar region, also below Pouparts ligament where there was crepitation. Thorax, heart and right lung normal left pleural cavity partially filled with pus of a very offensive odor, and a dark reddish color. The ribs corresponding to the fluid were hollowed out and covered with brittle calcareous plates. The cæcum and vermiform appendix was firmly bound to the sheath of the psoas muscle, which latter was much distended. On cutting into the psoas muscle a pulpy mass of greenish color and fecal odor oozed out. The abscess extended below Pouparts ligament. Cæcum and vermiform appendix healthy. No disease of the vertebræ. A slight congestion visible in mucous membrane of small intestines. Liver normal, kidneys normal, no communication through or under the pillars of the diaphragm with the abdomen.

Dr. Bigelow said he had a case somewhat similar two years ago. A man with dullness under the left scapula, and after a while a swelling on the inner side of the left thigh. Dr. Van Derveer saw the case with him and aspirated the abscess several times, drawing off a quantity of pus. The case finally recovered.

Dr. Boyd said cases were reported where there was communication under the pillars of the diaphragm, and in a case he saw the post-mortem of in New York, there was almost a complete calcareous casing of the affected chest.

Dr. Devol said he had seen several abscesses of the lungs. Had a patient where pus was expectorated, and the physician which had been in attendance said it was an abscess of the liver. The Doctor thought many abscesses find their way to the nearest opening. The first subject he had ever seen had an abscess of the lungs, which discharged itself between the vertebræ.

